# The Present Position of the Surgery of Malignant Growths

**Published:** 1911-12

**Authors:** Charles A. Morton

**Affiliations:** Professor of Surgery in the University of Bristol. Senior Surgeon to the General Hospital, and the Royal Hospital for Sick Children and Women


*"Scire est nescire, nisi id me
Scire alius sciret."*

DECEMBER, 1911.

### THE PRESENT POSITION OF THE SURGERY OF MALIGNANT GROWTHS.

The Presidential Address, delivered on October 11th, 1911, at the opening of the Thirty-eighth Session of the Bristol Medico-Chirurgical Society.

BY

CHARLES A. MORTON, F.R.C.S.,

*Professor of Surgery in the University of Bristol.*
*Senior Surgeon to the General Hospital, and the Royal Hospital for Sick Children and Women.*

---

It is often difficult to say anything worth saying when one is expected to give an address, and one's utterance is not the outcome of the ideas which one wishes to express. I do not wish simply to interest you with a subject which would be of no value when we had considered it, nor do I think I ought to make this address an opportunity for a new communication on a technical subject. I thought perhaps I should not be wasting your time if I spoke of the present position of the surgery of malignant growths. In looking through the subjects which past Presidents of this Society have taken for their addresses, I find that more than twenty years ago Mr. Dobson gave an address on some "Surgical Aspects of Carcinoma," and I

felt that after the lapse of twenty years the subject might well be again considered.

As the years go by surgeons more and more become impressed with the need for very extensive removal in dealing with a malignant tumour; they remove the surrounding parts more widely, and they follow the glands farther and farther away from the primary growth. I might give you illustrations of this in connection with the removal of malignant growths from almost all parts. Most surgeons now remove the great pectoral muscle in excising a cancerous breast, and some of us go farther, and as a routine practice clear out the supra-clavicular glands as well as the axillary, whether we can feel them to be enlarged or not, and in so doing sometimes remove glands already cancerous, but which could not be felt to be enlarged before operation. In operating for cancer of the uterus, much better results as to non-recurrence have been obtained by removing the cellular tissue around the lower segment of the uterus, and also the pelvic glands by the abdominal method of operating, than in the method of simply removing the uterus alone. Within a very short period of the removal of a cancer of the tongue, some of us now remove the set of glands in which recurrence is likely to take place, and do not wait for evidence of such recurrence.

Not only do surgeons remove the tissues more widely than they did a few years ago when operating for excision of malignant growths, but in some cases they remove the whole organ affected. We have been familiar with such a procedure in connection with cancer of the stomach for many years, but it is perhaps hardly so well known that the bladder has been removed in several cases after transplantation of the ureters. In certain situations in the bladder a malignant growth can be excised, together with a portion of the bladder wall; but if it involves the base extensively, resection of the whole organ may be the only possible way of removing it. In the female the ureters are transplanted into the vagina, and in the male the best plan seems to be to implant them on the skin of the loin at a preliminary operation. The mortality is high, but the chances of non-recurrence ought to be much better than after partial resection of the bladder wall.

To see cases of malignant disease recurrent after operation, and for which nothing further can be done, with their hopeless outlook—a condition almost always known to the patient—and in some cases the great suffering associated with such recurrence, is enough to stimulate us to take any conceivable step to prevent it. It should make us cut as far from the disease as we possibly can, without seriously endangering the patient's life, or sacrificing important organs or other useful parts; and in the case of malignant growth in a limb the question of amputation will have to be considered.

The great problem we have to face is how far we ought to incur this risk to life, and to what extent remove important structures to try and prevent recurrence. Let us consider the risk first. In operating for cancer of the breast we can cut very widely of the disease with very little risk, and every surgeon knows how by extensive undermining of the skin we can get it together in a way which would certainly much surprise anyone not accustomed to see such an operation; and the removal of the supra-clavicular glands adds very little risk indeed to the operation, provided that they are not fixed in the neighbourhood of the large veins at the root of the neck. The modern complete operation for cancer of the breast is necessarily a rather long procedure, but serious shock is quite the exception, and I have only had one death directly due to the operation in more than 110 cases,[1] and in almost half of these I have excised the great pectoral muscle as well as a large area of skin around the growth, and in a large number have also removed the supra-clavicular glands. In one case I removed at the same operation both cancerous breasts, both great pectorals, and cleaned out both axilla without marked shock in a woman 66 years of age.

[1] In the case of death directly due to the operation the wound was infected from an ulcerating growth, and the woman died from the sepsis. One woman died from the anæsthetic just as I commenced my incision. Two women died within a month of the operation after their wounds had healed, one of intrathoracic and one of abdominal disease, and in neither case was death due to the operation.

But when we come to deal with growths of the colon and rectum, extensive removal does add to the risk; and the question as to the advisability of extensive gland removal in these cases is a very important one, and in connection with it we have very seriously to consider what increased risk to life is involved in such a proceeding. In all operations for malignant disease there must be a group of glands liable to infection beyond the reach of surgery. Even if we excise the supra-clavicular glands in cancer of the breast, there may be infection of the mediastinal glands behind the upper end of the sternum, and in cancer affecting the colon the glands may be infected as high up as the cœliac axis. We can never, therefore, be absolutely certain of eradicating every trace of gland infection; but obviously the farther we can get above the disease the better our chance of preventing recurrence. It seems to me that a good rule to follow would be to remove every gland that might possibly be infected, until we reach a group the removal of which would be attended with distinct risk, and then to stay our hand. If we knew that the higher glands were already infected, we might perhaps wisely take an increased risk and remove them; but we must remember that often glands are found moderately enlarged in connection with malignant disease—at any rate, when the growth is in the colon—which microscopic examination does not show to be malignant. But such glands have not the marked hardness often present in cancer.

Now the removal of glands from above the growth, in cases of cancer of the colon, necessitates the removal of a much larger area of the colon (and sometimes also of part of the small intestine) than is really necessary to get well away from the disease in the bowel-wall, because in order to remove these glands a large area of the mesocolon has to be removed, and thus the vascular supply to a considerable length of bowel interfered with. Thus in cancer of the cæcum some surgeons now not only remove the cæcum, but also the ascending colon and the lower and of the ileum, because they think it well to remove the meso-colon containing the glands to which the lymphatics of the cæcum run, and in doing so they must remove the ileo-colic artery, on which the vascular supply of this extensive length of bowel depends. Obviously such extensive glandular excision is admissible if it does not add considerably to the shock of the operation. The mere removal of the greater length of bowel is not a serious thing. The bowel has only to be divided in two places, as in excision of the cæcum alone ; but there is extensive stripping up of the meso-colon containing the glands, and its high division and these conditions might increase shock. I think only further experience of this extensive gland removal can decide whether or not it should be carried out as a routine measure. I am now speaking of groups of glands either not enlarged at all or not to any extent. If one had to deal with probably malignant glands, then clearly it would be wise to take the risk, unless the glandular enlargment was so high up that it was quite a question whether even then malignant glands would not be left behind.

I should like to speak at greater length about the removal of one form of malignant growth of which I have had a fair amount of experience. I mean cancer of the rectum. It is very interesting to trace the evolution of the operation for its removal. Many of us remember the time when we dared not open the peritoneum. Occasionally it was opened by accident, but with much fear and trembling, and was immediately closed by suture. All cases of high cancer were abandoned to their fate. Then came Kraske's operation, and we deliberately opened the peritoneum and removed the high growths. Now I must say I have not found reason to be so dissatisfied with Kraske's operation as some surgeons are in respect of early recurrence, for several of my cases passed the three years' period without local recurrence, nor was my mortality from the operation great. But most of the local recurrences I have had have been in the bowel, and therefore it seems to me obviously desirable to remove a greater extent of bowel than can be done by a Kraske's operation, and I have now abandoned Kraske's operation in favour of the abdomino-perineal method. The whole rectum and pelvic colon is removed, and with it all the possibly infected glands in the meso-rectum and meso-colon. I am quite certain of this, that it is never wise to undertake any operation for cancer of the rectum without opening the abdomen to first discover the extent of the disease. You may find secondary nodules on the liver, which you could not feel through the abdominal wall; you may find, as I did on one occasion, a group of hard glands so high up in the meso-colon that it may be necessary to remove the meso-colon with the colon attached to it, as high as the origin of the inferior mesenteric artery, so as to get away the glands; or as I found in another case, a small infected gland may be present in the meso-colon near the bifurcation of the aorta, and this necessitated the resection of the base of the pelvic meso-colon, and as this contained the vessels supplying the pelvic colon, that had to be removed also, but such removal is a routine proceeding in the abdomino-perineal operation. Then again, from the interior of the abdomen you may discover fixation of the cancerous growth in the rectum to the bladder or uterus, and it then may or may not seem desirable to continue the operation, and remove such a growth, with perhaps a portion of the bladder or the whole uterus. Suppose you were to try and excise such a growth from the sacral aspect, what horrible difficulties you would soon get into. Even when working from the abdominal aspect, it may be most difficult to say whether the growth is only adherent to the bladder or uterus, and can be separated, or whether it really infiltrates them; so that if the growth is removed an extensive resection of the bladder wall must be made, or hysterectomy performed.

But by the abdominal method we sometimes find that a growth, apparently fixed on rectal examination, is not really fixed, and is removable. Now having opened the abdomen, and found that the growth is limited to the rectum, what advantage is there in excising it by the abdominal route? The great advantage seems to me to be that you can remove a greater length of the bowel above the growth, and clear out a large area of glands which are possibly if not evidently affected. This large area of glands could not possibly be removed by the sacral route, though you may suggest that a considerable length of bowel could be removed from above the growth by the sacral operation. But I find that it is impossible to do so without seriously interfering with the blood supply of the end of the colon which is dragged down, and this means that if you unite the divided ends, or even if you simply bring the cut end out below the cut surface of the sacrum, and make an artificial anus there, you will run great risk of sloughing of the end of the bowel. If you have united the ends the result of such sloughing is that the union gives way, that fæces are freely discharged through the wound for a time, and the wound is a very long time healing. In most cases union between the divided ends does eventually take place, but not in all ; and if you fix the end of the bowel to the skin below the sacrum, the part thus attached may slough away, and eventually only a track remains in scar tissue, leading from the surface to the bowel, which will tend greatly to contract, and this of course is very unsatisfactory. But if we divide the colon in the iliac fossa, and bring out the upper end there, we run no risk of sloughing if we are careful not to interfere with the blood supply by an unwise line of section through the meso-colon, and an iliac artificial anus is more readily attended to by the patient than a sacral one ; and, moreover, a pneumatic pad can be fixed over the opening by means of an abdominal belt.

But you may suggest that it is much better to make a union of the bowel, and secure the normal sphincter action in cases in which the growth is quite high up, and therefore in which a fair length of bowel can be left between the cut section and the anus. I have done this in most of my cases of Kraske's operation, and in some with a most excellent ultimate result, so that the patients had complete control after the operation, and I have only abandoned this method after much consideration.

It seems to me that looking at the fact that we can never be sure of the ultimate result in these cases (*i.e.* whether complete union will eventually take place), and that the bowel always gives way posteriorly soon after the operation, and a fæcal fistula forms, at any rate for a time, which makes convalescence very protracted, it is really better to cut very widely of the growth above, and remove all the section below, and clear out the glands in the way I have already mentioned, than to unite the divided ends even in very high cancerous growth; and in my opinion the recommendation of Mr. Miles that in an operation for cancer of the lower part of the rectum the levatores ani should be cut as far from the rectum as possible, is a very wise one, for recurrence takes place in some cases not in the end of the bowel, but in the surrounding tissues.

But how about the increased risk of these more extensive procedures—I mean high division of the bowel, with removal of the whole rectum and the gland area? No doubt there is a very decided increase in the risk, and the cause of that increased risk has been carefully considered. Some statistics have shown that it has been much greater in men than in women, and the explanation has been given that this is because the pelvis is deeper in men than in women, and therefore that operative manipulation is more difficult, and also that men stand all kinds of pelvic operations worse than women. Though a difference in the shape of the pelvis in males may account for some amount of increased difficulty in manipulation, this would not be sufficient to make a decided difference in the mortality, and it simply is not true to say that men stand pelvic operations worse than women. No doubt many more are done in women for obvious reasons, but when one has to operate on suppurative appendicitis in the pelvis of a man he does not specially suffer from shock. Really no satisfactory explanation of the remarkable difference in the mortality statistics of the abdomino-perineal operation for cancer of the rectum in men and women has been forthcoming, and it may turn out that it is merely an accident of the early statistics of the operation. With regard to the causes of the heavy mortality of the operation in both sexes, I have very carefully looked into the causes of death as given by Mr. Miles in a very valuable paper based on his experience of the operation in twenty-six cases (*Brit. Med. Journ.*, Oct., 1910). He lost ten out of the twenty-six cases, which is of course a very heavy mortality indeed. Only two were from shock at the time of the operation; but four died of "heart failure" a short time after, and though it is not easy to explain the exact method in which the operation brought about death in these cases, it seems reasonable to attribute the deaths to the operation, as Mr. Miles himself does.

Suppose, then, we consider all six cases as dying from a form of shock, this would seem to be the way in which so great a mortality is produced, for the other cases died from conditions which might be present in any intra-peritoneal bowel operation, and such an operation is Kraske's method of resection of part of the rectum.

During the last year I have had three cases of abdomino-perineal excision of the rectum for cancer, and all three were women, and in two the signs of severe shock came on during the operation; but by the use of immediate intravenous infusion I was able to finish the operation, and send them back to the ward with a fairly good pulse, nor did it fail later. In the third case there was no shock at the time of the operation, yet her pulse was not very good early next morning, but it very quickly improved with subcutaneous saline infusion. In the first case in which I did this operation (not one of those cases I have just mentioned) the growth seemed very advanced and quite fixed on rectal examination, and I opened the abdomen with the intention of performing simply a colostomy; but I found that from within the abdomen I could just move the mass of growth on the bony pelvis, and I removed it by the abdomino-perineal method; but it was an unusually extensive and difficult operation even for this method, and the man sank the day after the operation.

In another case I found the growth infiltrating the wall of the bladder, a condition which could only have been recognised by the abdominal operation, and in trying to separate the two organs, before it became evident that the condition was one of infiltration and not simply adhesion, I opened both the bladder and rectum, with fatal result. In yet another case, also in a male, I found it necessary to resect an unusual length of colon in order to get away a group of malignant glands situated as high up in the meso-colon as the origin of the inferior mesenteric artery; but in this case the growth was situated about the junction of rectum and pelvic colon, and I did the whole operation through his abdomen without any serious shock. In this case the lower —extra peritoneal—portion of the rectum was not removed.

In one of my last cases (a woman) I was not able to remove the growth even by the abdominal method, as it was too densely adherent to (possibly infiltrating) several coils of small intestine. This was not really a rectal growth, and could not be felt per rectum. It was a growth in the pelvic colon, but I intended to remove the whole pelvic colon and rectum if I had not discovered its fixation to small intestine. In one of the cases I have already referred to, the growth was also not strictly rectal, and could not be felt from the rectum. In both cases the growths were discovered in the pelvic colon when the abdomen was opened for the relief of obstruction.

My feeling at the present time is in favour of the abdomino-perineal operation in preference to Kraske's; but as I said just now, I have no reason to be greatly dissatisfied with Kraske's operation as regards recurrence, and I have had quite a low mortality with it.

Now although I have all along been advocating extension of our operative procedure for removal of cancer, I ought to say that I do not operate even so extensively as many surgeons in cancer of the rectum close to the anus, and I fear some surgeons would regard my present practice in these cases with very severe judgment; but I have had such good results as to non-recurrence after perineal excision of the lower portion of the rectum only, without opening the peritoneal cavity in such cases, that I still continue to perform this operation. It is a remarkably safe one, and by a preliminary abdominal incision the surgeon can always ascertain if the case is really suitable for it.

I will now turn to the consideration of quite another form of malignant growth. Some years ago I read a paper at a meeting of this Society, in which I advocated excision of periosteal sarcoma, and almost took the breath away from some members by what I am sure they considered my audacity and rashness; but I was only advocating what several German surgeons had already done with success, and since then Professor Alexis Thompson, of Edinburgh, has done the same operation in a case, details of which will no doubt be published presently, as the patient has already been shown at one of the medical societies in Edinburgh. I still hold the same opinion I did on the subject when I read that paper, *i.e.* that excision of a portion of the bone on which a periosteal sarcoma is growing might take the place of amputation in certain instances, and the experience of some surgeons in bone grafting has made this possible in cases in which otherwise it would not be so; but I feel that I ought to give this Society the result of my experience in one case in which I have employed the method since I published that paper. The case proves that a section of the bone an inch from the margin of the growth may not be far enough from it, and therefore limits our practice of the operation to cases in which the growth is very small, unless we can remove a large extent of bone, as in the fibula, or graft bone to take the place of the portion removed. In my case recurrence took place within a year on the shaft of the tibia, after resection of the lower end for periosteal sarcoma and division of the bone an inch above the upper level of the growth; but unfortunately, as generally happens in these cases even after amputation, the girl now has symptoms suggestive of intra-thoracic growth, three years after the resection of the bone and two after the amputation.

I must now say a few more words with regard to the subject of malignant growths in general. Time will not allow me to speak of measures of a non-operative kind, such as X-rays, radium, the administration of thyroid gland, Coley's fluid, and others used in non-operable cases. Perhaps I may just say that now and again they seem to check growth, and even occasionally to remove it; but they are all very unreliable, and cannot at the present time take the place of removal by the knife in suitable cases. But I wish to remind you of what is of the utmost importance with regard to success in the operative treatment of malignant growths, and what others have again and again insisted on, that we should be so well acquainted with their earliest signs that we could diagnose and treat them quite early. But, alas! early recognition does not depend on us in many cases. The patient pays no heed to the presence of the early stages of the disease because there is a widespread but mistaken idea that the commencement of a malignant growth must be very distressing. If the fact that this is not so could only be impressed on the minds of people who are not doctors we might get many more early cases. For instance, if it were recognised that a lump in the breast was possibly cancer, even though it was quite painless and the patient appeared to be in the best of health, many more cases would be operated on early.

And it would be well if we medical men recognised that diarrhœa which did not soon yield to treatment, or even simply a too-frequent action of the bowels, might be the only sign of early cancer in the rectum; that repeated attacks of abdominal colic, with alternating diarrhœa and constipation, might very likely be due to a growth in the colon; that what looked like a simple wart or ulcer on the tongue, especially if associated with a leucoplacic patch, might very likely be the early stage of epithelioma; that irregular uterine hemorrhage after the menopause was always a suspicious symptom of cancer of the uterus; and that symptoms of dyspepsia occurring after middle life which did not yield to treatment, especially if associated with marked wasting, might be the only symptoms of cancer of the stomach.

I wish I had time to consider the way in which patients are liable to die after operation for the removal of malignant growth; but this depends so much on the region of the growth, that it is quite impossible to give time to its consideration. But it is in a very careful study of the causes of death after these operations, as well as in early operation, that our hope lies for the future.

